# Comparisons between ethnic groups in hospitalizations for respiratory syncytial virus bronchiolitis in Israel

**DOI:** 10.1371/journal.pone.0214197

**Published:** 2019-04-01

**Authors:** Eias Kassem, Wasef Na'amnih, Amna Bdair-Amsha, Hazar Zahalkah, Khitam Muhsen

**Affiliations:** 1 Department of Pediatrics, Hillel Yaffe Medical Center, Hadera, Israel; 2 Department of Epidemiology and Preventive Medicine, School of Public Health, Sackler Faculty of Medicine, Tel Aviv University, Tel Aviv, Israel; The University of Hong Kong, CHINA

## Abstract

**Background:**

Ethnic disparities have been shown in respiratory syncytial virus (RSV) bronchiolitis. However, it is unclear whether such differences are related to access to care. We compared demographic and clinical characteristics of Arab and Jewish children hospitalized for RSV bronchiolitis in Israel, a country with universal health insurance.

**Methods:**

We reviewed the medical records of all children (n = 309) aged less than 24 months who were hospitalized with RSV between 2008 and 2011 in one medical center in Israel. Demographic, clinical, laboratory and radiological data were collected. The RSV antigen was identified using immunochromatography.

**Results:**

The annual incidence of RSV hospitalization was 5.4/1000 and 6.8/1000 among Arab and Jewish children, respectively. Arab patients were significantly younger and had significantly younger parents; most lived in low socioeconomic status towns (93.7% vs. 13.3%; p<0.001) and had more siblings (median 2 vs. 1; p = 0.01) compared to Jewish patients. Disease severity did not differ between the two ethnic groups (p = 0.3). The main predictors of severe illness were having pneumonia (adjusted odds ratio [OR] 3.86; 95% confidence intervals [CI] 1.87–7.97) and history of respiratory diseases (adjusted OR 3.89; 95% CI 1.22–12.38).

**Conclusions:**

The incidence of hospitalizations for RSV bronchiolitis tended to be higher among Jewish than Arab children, possibly due to differences in health care utilization patterns. Differences between the Jewish and Arab patients in demographic factors likely mirror differences between the groups in the general population. Pneumonia, and not ethnicity, affected the severity of RSV bronchiolitis.

## Introduction

Respiratory syncytial virus (RSV), a single-stranded RNA virus of the *Paramyxoviridae* family [1, 2], is the most common pathogen identified in young children hospitalized with acute lower respiratory infection; winter outbreaks of RSV are typical [3, 4]. Worldwide, RSV is estimated to cause 34 million episodes of acute lower respiratory infection annually in children younger than age five years[3].

Treatment of RSV bronchiolitis is mainly supportive [5]. Immuno-prophylaxis with RSV specific neutralizing monoclonal antibody, palivizumab, is recommended for high-risk groups such as premature infants and individuals with bronchopulmonary dysplasia [6–9]. RSV immuno-prophylaxis is used in many high-income countries, including the United States, European countries and Israel. However, the requirement of monthly administration and high cost are major limitations in developing countries [10]. Currently, there is no licensed active vaccine against RSV, but several are under development [10, 11]. Therefore, understanding the epidemiology and clinical course of RSV bronchiolitis is important to inform clinical and public health decision-making.

Ethnic disparities in RSV bronchiolitis have been documented [12–15]. A study from the United States showed higher rates of RSV hospitalizations in black children than white children aged 12–23 months; yet differences were not found in incidence rates in the first year of life, nor in the severity of disease [14]. It is not clear whether ethnic disparities in RSV bronchiolitis are related to access to care or to health care utilization patterns; or whether true ethnic differences exist in disease incidence. Studies from Israel have demonstrated a high burden from RSV bronchiolitis [16–18], with a higher incidence rate of RSV hospitalization in Arab Bedouin children, a small Muslim minority, compared to Jewish children in southern Israel [18]. The aim of the current study was to compare demographic and clinical characteristics of Arab and Jewish children hospitalized for RSV bronchiolitis in a region of Israel, a country with universal health insurance.

## Materials and methods

### Study design and population

The Israeli population comprises two main ethnic groups; Arabs (20%) and Jews (75%), while ~5% belong to other ethnicities [19]. We reviewed the data of all children aged less than 24 months, hospitalized with laboratory-confirmed RSV bronchiolitis between January 1, 2008 and December 31, 2011 at Hillel Yaffe Medical Center. This center is a 506-bed university-affiliated hospital that mainly serves the population of Hadera sub-district. About 450,000 people were living in this region during the study period, 54.3% of whom were Arabs. The numbers of live births among Arabs and Jews in Hadera sub-district were 3,944 and 3,598, respectively, in 2011 [19].

Demographic and clinical information was collected from medical records for the following variables: age in months (categorized as 0–5, 6–11, 12–23), sex, population group (Arabs or Jews), number of siblings and parental age. Socioeconomic status (SES) of the town of residence defined by the Israel Central Bureau of Statistics [20] was used as a proxy of SES. Children who lived in towns with SES ranks of 1–4 and 5–10 were classified as living in low and high SES communities, respectively.

Clinical information included birth weight (in grams), gestational age at birth (in weeks), hospitalization in a neonatal intensive care unit after birth, background morbidity, feeding (breastfeeding, formula, other), prior hospitalizations and emergency room visits. Information was collected on symptoms of current illness (fever [≥38°C], cough, runny nose, dyspnea, tachypnea, saturation and eating difficulties), diagnosis of pneumonia (by chest x-ray), results of laboratory tests and duration of hospital stay. A summative disease severity score was constructed based on the following variables: dyspnea, tachypnea, hypoxia (oxygen saturation<92), cough, fever and length of hospitalization. For each symptom, the child was “accredited” one point. Children with the median score or above were classified as having a severe illness. Chest x-ray findings were grouped as pneumonia vs. no pneumonia. Since chest X-ray was performed based on a physician's' referral and findings in physical examination, children with normal chest X-ray were grouped together with children who were not referred to chest X-ray as not having pneumonia.

### Laboratory methods

Patients’ respiratory samples (nasopharyngeal swabs or aspirates) were tested for the presence of RSV antigen at the laboratory of Hillel Yaffe Medical Center by immunochromatographic assay; BinaxNOW (Alere, Maine, USA). In children, the sensitivity and specificity of this kit were estimated at 90%-94% and 100%, respectively, compared to viral culture and/or reverse transcription polymerase chain reaction assay (RT-PCR) [21, 22].

### Statistical analysis

The incidence (per 1000 children) and 95% confidence intervals (CIs) of hospitalization for RSV bronchiolitis were calculated, using the number of cases of laboratory-confirmed RSV bronchiolitis in the numerator and the estimated population served by Hillel Yaffe Medical Center in the denominator. The size of the population aged <24 months was estimated as 40% of the population aged 0–4 years living in Hadera sub-district. We estimated that 80%-90% of the population in Hadera sub-district receives hospitalization services from Hillel Yaffe Medical Center. The estimated population of children aged <24 months was 26,310 and 24,588 of Arab and Jewish children, respectively, during the 48-month study period [19] (S1 Table).

Differences between Arab and Jewish patients in demographic and clinical characteristics were examined using the chi-square test or Fisher's exact test for categorical variables, Student's *t* test for continuous variables and Mann-Whitney U-test for variables with skewed distribution. We examined differences in disease severity according to population group and other factors, using the chi-square test and multivariable logistic regression models. The variables age and population group were selected a-priori to be included in the multivariable model. Clinically relevant independent variables that were associated with disease severity with p<0.2 in bivariate analysis were also included in the multivariable analysis in a stepwise manner. Collinearity between the independent variables was assessed using the variance inflation factor (VIF). Since the variables population group and residential SES were highly correlated (Phi coefficient 0.80, p<0.001), they were analyzed in separate models, we present both models with parameters of model fit such as Hosmer-Lemeshow goodness-of-fit test and Nagelkerke R^2^. Odds ratios (ORs) and 95% CIs were obtained from logistic regression models. Statistical significance was set at p<0.05. Data were analyzed using SPSS version 25 (IBM, Armonk, New York, USA).

### Ethical consideration

The study was approved by the Institutional Review Board (Helsinki) Committee of Hillel Yaffe Medical Center. Since this was a retrospective study, using archived medical records, an exempt from informed consent was granted by the Helsinki Committee.

## Results

Overall, 369 children aged less than 24 months hospitalized with bronchiolitis were tested for RSV, of whom 309 children (55.5% boys) were positive to RSV and met study inclusion criteria. The study sample included 166 (53.7%) Jewish patients and 143 (46.3%) Arab patients (S1 Table). Children were hospitalized for a median of 3 days (range: 1–34). Most patients (50.3%) were younger than age three months. Overall, the annual hospitalization rate for RSV bronchiolitis was 5.7 per 1000 children aged <24 months: 6.8 per 1000 and 5.4 per 1000 among Jewish and Arab children, respectively (relative risk 1.24 [95% CI 0.99–1.55]; p = 0.05).

**Fig 1:**
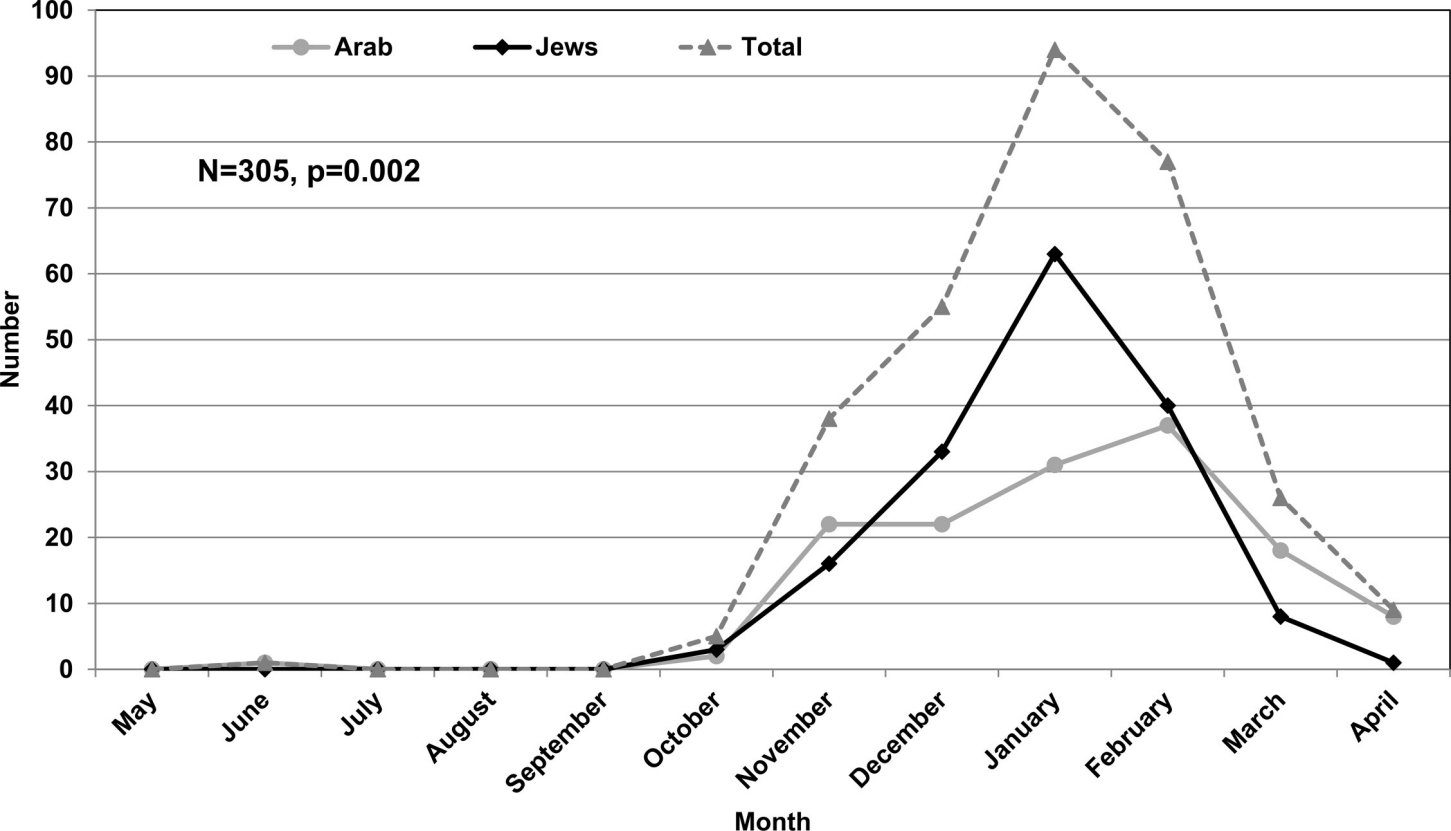
Number of monthly hospitalizations for RSV-bronchiolitis in children aged <24 months by population group (2008–2011). p = 0.002 for the difference between Arabs and Jews.

RSV bronchiolitis hospitalizations demonstrated typical winter seasonality. The rise in the number of cases began in November, peaked in January and decreased thereafter. Most (74.1%) cases occurred during December-February. The peak number of admissions was in January among Jewish children and February among Arabs (Fig 1).

### Sociodemographic and clinical characteristics by population group

The percentage of children younger than age 6 months was higher among Arab than Jewish patients. The majority (93.7%) of Arab children lived in low SES towns compared to 13.3% of Jewish patients (p<0.001). Parents of Arab children tended to be younger than parents of Jewish children (p<0.001 for maternal age, p = 0.03 for paternal age). The median number of siblings was higher among Arab than Jewish patients: 2 and 1, respectively (p = 0.01) (Table 1).

**Table 1:** Demographic characteristics of children hospitalized with RSV-bronchiolitis by population group*.

Variable	All, N = 309 N (%)	Arabs, N = 143 N (%)	Jews, N = 166 N (%)	P value
**Sex, male**	172 (55.5)	75 (52.4)	68 (47.6)	0.303
**Age, months**				
0–5	183 (60.2)	93 (66.0)	90 (55.2)	0.115
6–11	81 (26.6)	31 (22.0)	50 (30.7)	
12–23	40 (13.2)	17 (12.0)	23 (14.1)	
Missing	5 (1.6)	2 (1.4)	3 (1.8)	
**SES of place of residence**				
Low (1–4)	156 (50.6)	134 (93.7)	22 (13.3)	<0.001
High (5–10)	152 (49.4)	9 (6.3)	143 (86.7)	
Missing	1 (0.3)	0 (0.0)	1 (0.6)	
**Number of siblings** ^§^				
Median (min-max)	1 (0–7)	2 (0–6)	1 (0–7)	0.013
**Maternal age (years)** ^§^				
Mean (SD)	29.6 (5.4)	28.3 (5.5)	30.7 (5.1)	<0.001
**Paternal age (years)** ^§^				
Mean (SD)	33.3 (5.9)	32.4 (6.2)	34.1 (5.6)	0.029

* Max: maximum; min: minimum; RSV: respiratory syncytial virus; SD: standard deviation; SES: socioeconomic status.

^§^ Information was missing on number of siblings, maternal age and paternal age for 51 (16.5%), 44 (14.2%) and 48 (15.5%) children, respectively (S2 Table).

Information on the child's feeding practices was available for 107 (34.7%) children (S2 Table), showing that breastfeeding was more common among Arab than Jewish children: 37/54 (68.5%) vs. 18/53 (34.0%); p<0.001. Arab and Jewish children did not differ significantly in birth weight, gestational age at birth (in weeks), background morbidity and family history of asthma. Disease symptoms did not differ significantly between the groups, expect for cough and fever, which were less common in Arab than Jewish patients (Table 2).

**Table 2:** Clinical characteristics of children hospitalized with RSV-bronchiolitis by population group.

Variable	All (N = 309)	Arabs (N = 143)	Jews (N = 166)	P value
	N/total (%)	N/total (%)	N/total (%)	
**Health and medical history**				
Breastfeeding	55/107 (51.4)	37/54 (68.5)	18/53 (34.0)	<0.001
Family history of asthma	53/259 (20.5)	20/119 (16.8)	33/140 (23.6)	0.217
Median weight at birth (grams) (min-max)*	3080 (740–5505)	3095 (893–5505)	3100 (740–4925)	0.715
Gestational age at birth (weeks)				
<34	13/301 (4.3)	8/139 (5.8)	5/162 (3.1)	0.503
34–36	37/301 (12.3)	16/139 (11.5)	21/162 (13.0)	
≥37	251/301 (83.4)	115/139 (82.7)	136/162 (84.0)	
Background respiratory disorders	27/309 (8.7)	9/143 (6.3)	18/166 (10.8)	0.145
Congenital heart disease	14/309 (4.4)	9/143 (6.3)	5/166 (3.0)	0.164
Neurological defect	12/309 (3.9)	8/143 (5.6)	4/166 (2.4)	0.135
Illness in the last month before hospitalization	36/309 (11.6)	15/143 (10.5)	21/166 (12.6)	0.597
Past admission	46/309 (15.4)	22/143 (15.4)	24/166 (14.5)	0.873
**Symptoms, signs of current illness**				
Dyspnea	221/304 (72.5)	105/141 (74.5)	116/163 (71.2)	0.519
Strangulation	26/298 (8.7)	13/136 (9.6)	13/162 (8.0)	0.640
Cough	272/304 (89.2)	119/140 (85.0)	153/164 (92.7)	0.041
Fever (≥38°C)	189/303 (62.4)	77/139 (55.4)	112/164 (68.3)	0.024
Runny nose	155/298 (51.8)	65/135 (48.1)	89/163 (54.6)	0.295
Apathy	22/300 (7.3)	7/136 (5.1)	15/164 (9.1)	0.186
Tachypnea	262/303 (86.5)	123/139 (88.5)	139/164 (84.8)	0.344
Hypoxia (Saturation<92%)	29/297 (9.7)	14/136 (10.3)	15/161 (9.3)	0.777
Eating difficulty	117/302 (38.6)	57/139 (41.0)	60/163 (36.8)	0.479
Pneumonia by chest X-ray	66/309 (21.4)	28/143 (19.6)	38/166 (22.9)	0.479
Co-infection	84/309 (27.2)	41/143 (28.7)	43/166 (25.9)	0.610
Duration of hospital stay >4 days	138/304 (45.4)	69/140 (49.3)	69/164 (42.1)	0.248

Max: maximum; min: minimum; SD: standard deviation; RSV: respiratory syncytial virus.

* Data on birth weight were missing for 17 children.

### Factors associated with the severity of RSV among hospitalized children

The median score of disease severity was 4 (range: 1–6), both among Arab and Jewish children. Significant differences were not found in disease severity between Arabs and Jews, and between males and females. A positive association was shown between child's age and disease severity (p for trend 0.024). The percentage of children who lived in high SES towns was higher among those with severe disease vs. children with less severe disease, but the difference was not statistically significant (53.8% vs. 43.7%; p = 0.08). Children with severe disease more often had background respiratory disorders (13.3% vs. 2.9%; p = 0.001), co-infection (32.4% vs. 20.8%; p = 0.02), and a diagnosis of pneumonia than children with less severe disease (Table 3).

**Table 3:** Factors associated with the severity of RSV-bronchiolitis in hospitalized children aged less than 24 months.

Variable	Severe disease (N = 173)	Less severe disease (N = 136)	OR (95% CI)	P value
	N (%)	N (%)		
**Population group**				
Arabs	75 (43.4)	68 (50.0)	0.77 (0.49–1.20)	0.245
Jews	98 (56.6)	68 (50.0)	Reference	
**Sex**				
Male	99 (57.2)	73 (53.7)	1.08 (0.84–1.39)	0.533
Female	74 (42.8)	63 (46.3)	Reference	
**Age, months**			df = 2	0.026
0–5	93 (54.1)	90 (68.2)	0.39 (0.18–0.83)	0.015
6–11	50 (29.1)	31 (23.5)	0.61 (0.26–1.39)	0.244
12–23	29 (16.9)	11 (8.3)	Reference	P trend 0.008
Missing	1 (0.6)	4 (2.9)		
**Residential SES**				
Low SES (1–4)	80 (46.2)	76 (56.3)	0.67 (0.43–1.05)	0.080
High SES (5–10)	93 (53.8)	59 (43.7)	Reference	
Missing	0 (0.0)	1 (1.6)		
**Illness in the last month** ^§^	24 (13.9)	12 (8.8)	1.66 (0.80–3.46)	0.170
**Past admission** ^§^	29 (16.8)	17 (12.5)	1.41 (0.74–2.69)	0.296
**Co-infection** ^§^	56 (32.4)	28 (20.6)	1.85 (1.09–3.12)	0.021
**Chest X-ray**				
Pneumonia	53 (30.6)	13 (9.6)	4.18 (2.17–8.06)	<0.001
No Pneumonia*	120 (69.4%)	123 (90.4%)	Reference	
**Background respiratory disorders** ^§^	23 (13.3)	4 (2.9)	5.06 (1.70–15.00)	0.001
**Congenital heart disease** ^§^	8 (4.6)	6 (4.4)	1.05 (0.36–3.10)	0.929

CI: confidence interval; df: degrees of freedom; OR: odds ratio; RSV: respiratory syncytial virus; SES: socioeconomic status.

^§^ Reference category = not having the condition.

* No pneumonia by chest X-ray or by clinical judgment (chest X-ray was not ordered by a physician).

The variable child's age was positively correlated with having background respiratory disease (Phi correlation coefficient 0.26, p<0.001): 5/183 (2.7%) infants aged 0–5 months had a background respiratory disease, compared to 15/81 (18.5%) and 7/40 (17.5%) in those aged 6–11 and 12–23 months.

In a multivariable analysis that included 303 children (Model 1 Table 4), the associations of child's age and SES with disease severity were not statistically significant (p = 0.19, and 0.14, respectively). Background respiratory diseases and pneumonia (confirmed by chest X-ray), were strongly associated with a 4-fold increased risk for severe disease (adjusted OR 3.89 [95% CI 1.22–12.38]; p = 0.02 and adjusted OR 3.86 [95% CI 1.87–7.97] p<0.001, respectively). A second model that included the variable population group instead of residential SES showed similar results, as well as no significant difference in disease severity according to population group (Model 2 Table 4). VIF values ranged between 1.01–1.065, suggesting no collinearity between the independent variables.

**Table 4:** Multiple logistic regression models for factors associated with RSV severity.

	Model 1		Model 2	
Variable	Adjusted OR (95% CI)	P value	Adjusted OR (95% CI)	P value
**Age, months**	df = 2	0.187	df = 2	0.343
0–5	0.46 (0.20–1.06)	0.068	0.55 (0.25–1.23)	0.147
6–11	0.55 (0.22–1.36)	0.196	0.65 (0.27–1.56)	0.335
12–23	Reference		Reference	
**Residential SES** **Low (1–4) vs. high (5–10)**	0.69 (0.42–1.12)	0.136	Not included	
**Population group (Jews vs. Arabs)**	Not included		1.20 (0.74–1.95)	0.449
**Illness in the last month before hospitalization** **(yes vs. no)**	1.57 (0.72–3.44)	0.256	1.62 (0.75–3.53)	0.223
**Background respiratory disorders (yes vs. no)**	3.89 (1.22–12.38)	0.022	4.05 (1.28–12.79)	0.017
**Co-infection (yes vs. no)**	1.13 (0.61–2.08)	0.698	1.20 (0.66–2.20)	0.547
Chest X-ray-pneumonia (yes vs. no)*	3.86 (1.87–7.97)	<0.001	3.54 (1.76–7.15)	<0.001

Nagelkerke R^2^ model 1 = 0.161, Nagelkerke R^2^model 2 = 0.146

CI: confidence interval; df: degrees of freedom; OR: odds ratio; RSV: respiratory syncytial virus; SES: socioeconomic status.

* No pneumonia by chest X-ray or by clinical judgment (chest X-ray was not ordered by a physician)

## Discussion

In a setting of universal health care insurance, we assessed ethnic differences in hospitalizations for RSV bronchiolitis in children aged less than 24 months. The incidence of hospitalizations for RSV bronchiolitis was higher among Jewish than Arab children. Arab and Jewish patients differed in sociodemographic factors but not in the clinical characteristics of their illness. Arab patients tended to be younger than Jewish patients. Parents of Arab patients also tended to be younger than parents of Jewish patients. Arab patients more often lived in communities of lower SES than Jewish patients and had more siblings. These differences likely reflect the existing general sociodemographic variation between the two population groups. Indeed, in Israel in general, and in the examined region in particular, the Arab population is younger than the Jewish population (median age; 21.8 vs. 33.2, respectively), has larger families and is of lower SES, as reflected by average income and education [19]. Although information on the child's feeding practices was available for only 35% of the sample, we found that the Arab patients were more often breastfed than the Jewish patients (68.5% vs. 34.0%; p<0.001). This observation is in agreement with a previous study that showed that the intention for exclusive breastfeeding was higher among Muslim than Jewish women [23], and with a national survey of children from birth until age 2 years, which showed higher breastfeeding rates among Arab than Jewish mothers [24].

Despite the above-mentioned sociodemographic disparities between Arab and Jewish RSV patients, overall, we found no significant differences in clinical characteristics or in the severity of RSV bronchiolitis between hospitalized Arab and Jewish children. Studies from the United States have shown that children belonging to minorities and those of lower SES were more likely to present with bronchiolitis to the emergency department and to subsequently be admitted compared to the general population [25, 26]. In contrast, other US studies [13, 14] did not find significant differences between black and white children in regard to RSV hospitalizations. Previous studies on SES disparities in RSV bronchiolitis showed higher rates in children from low vs. high SES, and in children from rural regions with limited access to care [13, 27–29]. In Australia, the risk of hospitalization due to RSV bronchiolitis was shown to be higher in Indigenous compared to non-Indigenous infants, and this was largely attributed to factors related to lower SES [27]. In both Indigenous and non-Indigenous children, better SES was related to lower risk for hospitalization due RSV bronchiolitis [30].

In southern Israel, Bedouin children are at higher risks of hospitalizations for infectious diseases, as well as for RSV bronchiolitis in early childhood, compared to Jewish children [18, 31]. The Bedouin population in southern Israel differs from the Arab population in the rest of Israel. About 50% of the Bedouin population lives in unrecognized villages and tribes, with limited access to health care facilities. This differs from other regions of Israel. Although in the current study, the SES was lower in the towns and villages where the Arabs compared to the Jews resided, all the residential areas have a basic and similar health care infrastructure. Considering the studies from various populations described above, the higher incidence of hospitalizations for RSV bronchiolitis in the Jewish population in our study likely reflects differences in referral or health care utilization patterns, or a combination of these factors.

In both population groups in the current study, most patients were under age 6 months (66% and 55% of the Arab and Jewish patients, respectively). Similarly, a review of studies of RSV hospitalizations reported that 49% to 70% of hospitalized infants with RSV are below age 6 months, and 66% to 100% younger than 1 year [32].

No significant differences were found between patients with more and less severe disease according to population group, sex, SES, birth week, birth weight, number of siblings and breastfeeding. The lack of correlation between these factors and severe bronchiolitis is consistent with findings reported by Papoff et al. [33]. We observed that children with more severe RSV were more likely to be admitted with evidence of lower respiratory involvement (e.g., higher frequency of pneumonia or an abnormal X-ray) and with background respiratory disorders, compared to those with less severe RSV. This finding is similar to that reported by Halasa et al. [34]. We found that co-infection is more likely to cause RSV severity in a bivariate analysis. Several studies have shown that co-infection results in higher hospitalization rates and longer hospital length of stay. However, up to one quarter of patients hospitalized with bronchiolitis were found to be co-infected with multiple agents [35, 36]. Unlike previous reports [33, 34, 37] we found that the percentage of children younger than age 6 months was significantly higher among those with less severe than more severe disease in bivariate analysis. A positive association between age and having background respiratory disease (Phi correlation coefficient 0.26, p<0.001). The association between age and disease severity became non-statistically significant in multivariable models after adding the variable background respiratory diseases, thus suggesting that age might be a marker of background diseases. Prevention of RSV disease in young children may ultimately be possible with active immunization or maternal immunization [38, 39]. The only currently available preventive measure for RSV disease is palivizumab, which reduces the risk of hospitalization caused by RSV in high-risk children [40]. Most of the children hospitalized with RSV in our study were full-term and otherwise healthy children, who did not meet the criteria for receiving the palivizumab vaccine. The latter is recommended only for a small proportion of infants who are born prematurely or who have exacerbating comorbidities, and thus this vaccination has little impact on overall RSV hospitalization rates. Therefore, the development of effective general preventive strategies such as infant or maternal vaccination or antiviral therapies specific for RSV is needed for all young infants, to reduce the burden of RSV hospitalizations.

Our study has limitations. First, not all children hospitalized due to respiratory illnesses underwent RSV testing; hence, the incidence of RSV bronchiolitis may have been underestimated. However, the decision to test for RSV is driven by clinical characteristics and not by ethnic group. Moreover, it is estimated that the study center serves 80%-90% of children residing in the region, therefore the representativeness of the study sample is high. Second, we used data from medical records over a 4-year study period. Differences could exist between physicians in obtaining and reporting medical information; however, such differences would likely not be related to children's ethnic background. Information on breastfeeding was available for 35% of the children, but this rate was similar among Arab (38%) and Jewish children (32%), (p = 0.2). Missing information on other variables was overall low, and the percentage of children with missing information was similar between Jewish and Arab children, the impact on the validity of our findings is limited.

The strengths of our study include the use of multi-year data, which yielded robust incidence estimates, in addition to the availability of comprehensive clinical and demographic information. This enabled broad comparisons between Arab and Jewish RSV patients. Lastly, since our study was conducted in a setting with universal health insurance, we were able to control for this factor as a potential contributor to ethnic disparities in RSV bronchiolitis.

In conclusion, the incidence of hospitalizations for RSV bronchiolitis tended to be higher among Jewish than Arab children in a setting of universal health insurance, likely reflecting health care utilization patterns. Infants less than 6 months of age comprised the majority of children hospitalized for RSV bronchiolitis, in both population groups. Differences in demographic characteristics between the Arab and Jewish children with RSV likely mirror differences between Arabs and Jews that exist in the general population, but they do not seem to affect disease severity. Most patients were ineligible for receiving the palivizumab vaccine; hence, the development of effective general preventive measure targeting all infants such as infant or maternal vaccination is warranted to reduce the burden of RSV bronchiolitis.

## Supporting information

S1 Table Number of children aged <24 months hospitalized with RSV bronchiolitis (and population size) by year, population group and month. (XLSX)

S2 Table Number (and percentage) of children with missing data on the study variables by population group. (DOCX)
