# Supplementary material for: Comparisons between ethnic groups in hospitalizations for respiratory syncytial virus bronchiolitis in Israel
Source: PLoS One. 2019 Apr 1;14(4):e0214197. doi: 10.1371/journal.pone.0214197 (PMC6443173; doi:10.1371/journal.pone.0214197)
Supplement: S2 Table — (DOCX) [file pone.0214197.s002.docx]

**S2 Table. Number (and percentage) of children with missing data on the study variables by population group**

| **Variable** | **All, N=309**  **N (%)** | **Arabs, N=143**  **N (%)** | **Jews, N=166**  **N (%)** |
| --- | --- | --- | --- |
| **The child's Age** | 5 (1.6) | 2 (1.4) | 3 (1.8) |
| **Socioeconomic status of place of residence** | 1 (0.3) | 0 (0.0) | 1 (0.6) |
| **Weight at birth** | 17 (5.5) | 9 (6.3) | 7 (4.2) |
| **Gestational age at birth (in weeks)** | 8 (2.6) | 4 (2.8) | 4 (2.4) |
| **Dyspnea** | 5 (1.6) | 2 (1.4) | 3 (1.8) |
| **Strangulation** | 11 (3.6) | 7 (4.9) | 4 (2.4) |
| **Cough** | 5 (1.6) | 3 (2.1) | 2 (1.2) |
| **Fever (>38 °C)** | 6 (1.9) | 4 (2.8) | 2 (1.2) |
| **Runny nose** | 11 (3.6) | 8 (5.6) | 3 (1.8) |
| **Apathy** | 9 (2.9) | 7 (4.9) | 2 (1.2) |
| **Tachypnea** | 6 (1.9) | 4 (2.8) | 2 (1.2) |
| **Hypoxia (Saturation<92%)** | 12 (3.9) | 7 (4.9) | 5 (3.0) |
| **Eating difficulty** | 7 (2.3) | 4 (2.8) | 3 (1.8) |
| **Duration of hospital stay >4 days** | 5 (1.6) | 3 (2.1) | 2 (1.2) |
| **Number of siblings** | 51 (16.5) | 35 (24.5) | 16 (9.6) |
| **Maternal age** | 44 (14.2) | 18 (12.6) | 26 (15.7) |
| **Paternal age** | 48 (15.5) | 19 (13.3) | 29 (17.5) |
| **Family history of asthma** | 50 (16.2) | 24 (16.8) | 26 (15.7) |
| **Child's feeding practices** | 202 (65.4) | 89 (62.2) | 113 (68.1) |
